# Genome Sequence of *Parvimonas micra* Strain A293, Isolated from an Abdominal Abscess from a Patient in the United Kingdom

**DOI:** 10.1128/genomeA.01025-13

**Published:** 2013-12-05

**Authors:** Mia Yang Ang, David Dymock, Joon Liang Tan, Ming Hang Thong, Qin Kai Tan, Guat Jah Wong, Ian C. Paterson, Siew Woh Choo

**Affiliations:** Genome Informatics Research Laboratory, High Impact Research Building (HIR) Building, University of Malaya, Kuala Lumpur, Malaysiaa; Department of Oral Biology and Biomedical Sciences, University of Malaya, Kuala Lumpur, Malaysiab; School of Oral and Dental Sciences, University of Bristol, Bristol, United Kingdomc; Oral Cancer Research and Coordinating Centre, Faculty of Dentistry, University of Malaya, Kuala Lumpur, Malaysiad

## Abstract

*Parvimonas micra* is an important oral microbe that has the ability to grow and proliferate within oral biofilms and is involved in periodontal disease, leading to gingival bleeding, gingival recession, alveolar bone loss, and tooth mobility. However, occasionally these normally oral pathogens can cause infections at other sites in the body. We present the genome sequence of *Parvimonas micra* strain A293, a smooth *Parvimonas micra* strain isolated from an abdominal abscess from a patient at Barts Hospital, London, United Kingdom.

## GENOME ANNOUNCEMENT

*Parvimonas micra*, formerly known as *Peptostreptococcus micros* or *Micromonas micros* (1), is an anaerobic, Gram-positive, and non-spore-forming bacterial species (2). *P. micra* is often found within subgingival dental plaque and causes periodontal problems such as periodontitis (3) and periodontal decline in elderly individuals (4). This species is most commonly isolated from the human oral cavity. However, under certain conditions, such as immunosuppression, *P. micra* may become more invasive and cause infections in other parts of the body. Therefore, *P. micra* is also categorized as one of the members of endogenous bacteria in the human body (5).

We have sequenced the draft genome of a smooth *P. micra* strain isolated from an abdominal abscess from a patient in the United Kingdom. The genome of *P. micra* strain A293 was shotgun sequenced using the Illumina HiSeq 2000 paired-end sequencing platform. The 17,592,576 raw reads generated from this platform in this study were preprocessed using PRINSEQ-lite version 0.20.1 to remove duplicated reads and poor-quality reads (6). The preprocessed reads were *de novo* assembled using the CLC Genomics Workbench, which resulted in 49 contigs, with an *N*_50_ contig size of 122,624 bp. The contig size ranged from 539 bp (shortest contig) to 407,487 bp (longest contig). The genomic size of this assembly is 1,680,794 bp, with a low G+C content of 28.3%.

The sequenced genome was annotated using the Rapid Annotation Subsystem Technology (RAST) pipeline (7). RAST predicted 1,560 coding sequences (CDS) and 46 RNAs (40 tRNAs and 6 rRNAs). RAST functional annotation of the predicted protein-coding genes showed 137 genes involved in protein metabolism, 60 in carbohydrate metabolism, 86 in amino acid and derivatives synthesis, 58 in DNA metabolism, and 89 in RNA metabolism; 62 genes responsible for nucleoside and nucleotide formation; and 3 genes associated with phages, prophages, transposable elements, and plasmids. The RAST server also revealed the top three closest neighbors of *P. micra* strain A293, which were *P. micra* ATCC 33270, *Finegoldia magna* ATCC 29328, and *Finegoldia magna* ATCC 53516.

To predict the prophages in the genome of *P. micra* strain A293, we used the phage Search Tool (PHAST) (8). PHAST analysis revealed two putative prophage regions in *P. micra* strain A293. One of the prophages is an intact/complete prophage, whereas the other one is an incomplete prophage, as predicted by the PHAST software. The intact prophage consists of 54 putative CDS, and the incomplete prophage contains only 20 CDS. The complete and incomplete prophages have G+C contents of 27.7% and 24.5%, respectively.

In conclusion, we report the genome of *P. micra* strain A293. The addition of this new member of *P. micra* may enhance our insight into the biology, evolution, diversity, and pathogenicity of this human oral pathogen.

### Nucleotide sequence accession number.

This whole-genome shotgun project has been deposited at DDBJ/EMBL/GenBank under the accession AXUQ00000000.

## References

[1] Tindall B Euzéby J 2006 Proposal of *Parvimonas* gen. nov. and *Quatrionicoccus* gen. nov. as replacements for the illegitimate, prokaryotic, generic names *Micromonas* Murdoch and Shah 2000 and *Quadricoccus* Maszenan et al. 2002, respectively. Int. J. Syst. Evol. Microbiol. 56:2711–2713 17082417 10.1099/ijs.0.64338-0

[2] Murdoch D Collins M Willems A Hardie J Young K Magee J 1997 Description of three new species of the Genus *Peptostreptococcus* from human clinical Specimens: *Peptostreptococcus harei* sp. nov., *Peptostreptococcus ivorii* sp. nov., and *Peptostreptococcus octavius* sp. nov. Int. J. Syst. Bacteriol. 47:781–787

[3] Rams TE Feik D Listgarten MA Slots J 1992 *Peptostreptococcus* micros in human periodontitis. Oral Microbiol. Immunol. 7:1–6 1528618 10.1111/j.1399-302x.1992.tb00011.x

[4] Swoboda JR Kiyak HA Darveau R Persson GR 2008 Correlates of periodontal decline and biologic markers in older adults. J. Periodontol. 79:1920–1926 18834247 10.1902/jop.2008.080005

[5] Narayanan LL Vaishnavi C 2010 Endodontic microbiology. J. Conserv. Dent. 13:233–239 21217951 10.4103/0972-0707.73386 PMC3010028

[6] Schmieder R Edwards R 2011 Quality control and preprocessing of metagenomic datasets. Bioinformatics 27:863–864 21278185 10.1093/bioinformatics/btr026 PMC3051327

[7] Aziz RK Bartels D Best AA DeJongh M Disz T Edwards RA Formsma K Gerdes S Glass EM Kubal M Meyer F Olsen GJ Olson R Osterman AL Overbeek RA McNeil LK Paarmann D Paczian T Parrello B Pusch GD Reich C Stevens R Vassieva O Vonstein V Wilke A Zagnitko O 2008 The RAST server: rapid annotations using subsystems technology. BMC Genomics 9:75 18261238 10.1186/1471-2164-9-75 PMC2265698

[8] Zhou Y Liang Y Lynch KH Dennis JJ Wishart DS 2011 PHAST: a fast phage search tool. Nucleic Acids Res. 39:W347–W352 21672955 10.1093/nar/gkr485 PMC3125810

